# Pulmonary wave intensity analysis to assess ventriculo-arterial interactions in cardiogenic shock

**DOI:** 10.1016/j.jhlto.2026.100528

**Published:** 2026-02-26

**Authors:** Sara L. Hungerford, Kay D. Everett, Edmund Lau, Daniel Burkhoff, Navin K. Kapur, Gaurav Gulati

**Affiliations:** a The CardioVascular Center Tufts, Boston, MA; b Faculty of Health and Medicine, The University of Sydney, Sydney, New South Wales, Australia; c Royal North Shore Hospital, Sydney, New South Wales, Australia; d Cardiovascular Research Foundation, New York, NY

**Keywords:** Cardiogenic shock, Pulmonary arterial, Pulmonary hypertension, Pulmonary vascular hemodynamics, Pulmonary vascular resistance, Right ventricular, WIA

## Abstract

**Background:**

Pulmonary wave intensity analysis (WIA) uses pressure and velocity measurements to characterize the type, direction, and timing of energy waves within the pulmonary circulation. Its application to assess ventriculo-arterial (VA) interactions in patients with cardiogenic shock (CS) receiving mechanical circulatory support (MCS) has not been previously described. We hypothesized that forward wave intensity would be reduced in CS, with preserved or augmented reflected wave intensity and/or speed.

**Study Design and Methods:**

In this prospective cohort study, 19 patients with CS requiring MCS were compared with 10 patients with normal pulmonary artery (PA) hemodynamics. PA pressure was measured via right heart catheterization (RHC), and PA flow via Doppler transthoracic echocardiography (TTE). WIA was derived from the product of pressure and velocity change. Statistical significance was defined as *p* < 0.05.

**Results:**

We studied 19 CS patients (54 ± 9 years, 17 male) and 10 controls (52 ± 16 years, 7 male). Compared with controls, CS patients had lower LVEF (16 ± 7 vs 50% ± 15%), lower RV FAC (21 ± 11 vs 39% ± 13%), and higher PCWP (25 ± 10 vs 9 ± 4 mmHg; all *p* < 0.01). CS was associated with more negative net wave intensity (–0.05 ± 0.07 vs –0.02 ± 0.02) and higher wave speed (7.1 ± 7.4 vs 1.8 ± 1.0 cm/s; both *p* < 0.05). Following MCS initiation, peak backward compression wave timing was delayed (0.13 ± 0.07 vs 0.17 ± 0.05 s, *p* = 0.048). IABP support was associate with increased forward decompression wave magnitude (*p* = 0.04) and reduced reflection index (*p* = 0.046), whereas Impella 5.5 delayed backward compression wave return (*p* = 0.03), without consistent changes in mPAP or PVR. WIA parameters demonstrated associations with LVEDV (BDW: *R²* = 0.13, *p* = 0.02), LA volume (BCW: *R²* = 0.23, *p* < 0.01), RV EDA (BCW: *R²* = 0.31, *P* < 0.01), TAPSE (*R²* = 0.08, *p* = 0.08), and inotrope dose (dobutamine: NWI *R²* = 0.30, *p* = 0.02; milrinone: TTP *R²* = 0.37, *p* < 0.01).

**Conclusions:**

In CS, net wave intensity was more negative, and wave speed increased. MCS was associated with changes in forward and reflected wave characteristics, consistent with altered RV-pulmonary arterial coupling. WIA parameters were associated with structural and functional cardiac measures and reflected physiologic changes not consistently captured by conventional hemodynamic or echocardiographic indices. These exploratory findings warrant validation in larger prospective cohorts.

## Background

Pulmonary wave intensity analysis (WIA) is a time-domain method that uses pressure and velocity measurements to assess the intensity, direction, type, and timing of waves.^1^ Traveling wavefronts represent energy transmission between the right ventricular (RV) and pulmonary arterial (PA).^2^ Forward-traveling waves originate from the RV, whilst backward-traveling waves are reflection from the distal pulmonary vasculature.^2^ Wavefronts are typically classified into 4 types: (1) forward compression waves that *increase* pressure and velocity, (2) forward decompression waves that *decrease* pressure and velocity, (3) backward compression waves that *increase* pressure but *decrease* velocity, and (4) backward decompression waves that *decrease* pressure but *increase* velocity.^2^ WIA also quantifies wave speed and reflected wave timing (i.e., the point in systole when the reflected wave returns from the distal pulmonary vasculature).

Whereas pulmonary WIA has been extensively studied in conditions such as PA hypertension and chronic thromboembolic disease,^3^, ^4^, ^5^ its application in those with pulmonary hypertension (PH) from left-sided heart disease, specifically those with cardiogenic shock (CS) requiring mechanical circulatory support (MCS), remains poorly defined. Limited studies suggest that disruption of wave hemodynamics may play an important role in modulating ventricular afterload and coronary perfusion in CS.^6^, ^7^ Theoretically, CS might lead to a reduction in forward-traveling wave intensity (due to poor contractility of the left [LV] or right ventricle [RV]), whereas backward-traveling wave intensity, reflected wave timing and/or speed may remain unchanged or increased (owing to sympathetic neurohormonal activation or pulmonary venous congestion).

In this context, we sought to measure PA wave propagation in subjects with heart failure-related CS undergoing MCS. We hypothesized that for subjects with CS, forward wave intensity would be markedly reduced, while reflected wave intensity, timing, and/or speed would remain relatively unchanged, leading to an overall negative net wave intensity.

## Methods

### Study populations

We screened all post-heart transplant and CS patients over a 12-month period who underwent diagnostic right heart catheterization (RHC). Ten control subjects were recruited from among the post-heart transplant population at our institution, as these patients were most likely to be receiving clinically indicated RHC and have normal PA hemodynamics. Control subjects were required to be >18 years old, >6 months from their transplant, and have normal hemodynamics, defined as right atrial pressure (RAP) <10 mmHg, pulmonary artery systolic pressure (PASP) <35 mmHg, mean pulmonary artery pressure (mPAP) <25 mmHg, pulmonary capillary wedge pressure (PCWP) <20 mmHg, and cardiac index (CI) > 2.2 L/min/m^2^, without the use of pulmonary vasodilator therapy.

Nineteen subjects were enrolled in the CS cohort and required MCS. CS was defined as a CI < 2.2 L/min/m^2^ and any one of the following in the 24 hours prior to enrollment: systolic blood pressure <90 mmHg, lactate >2.0 mEq/L, 30% rise in creatinine from baseline, or 50% rise in alanine aminotransferase. CS cohort subjects were required to have either an intra-aortic balloon pump (IABP) or Impella 5.5 implant planned within 24 hours of enrollment. The choice of MCS device was made by the treating physician based on the patient’s clinical context. CS subjects were excluded if they were mechanically ventilated or had a cardiac arrest within the prior 12 hours.

**Figure 1:**
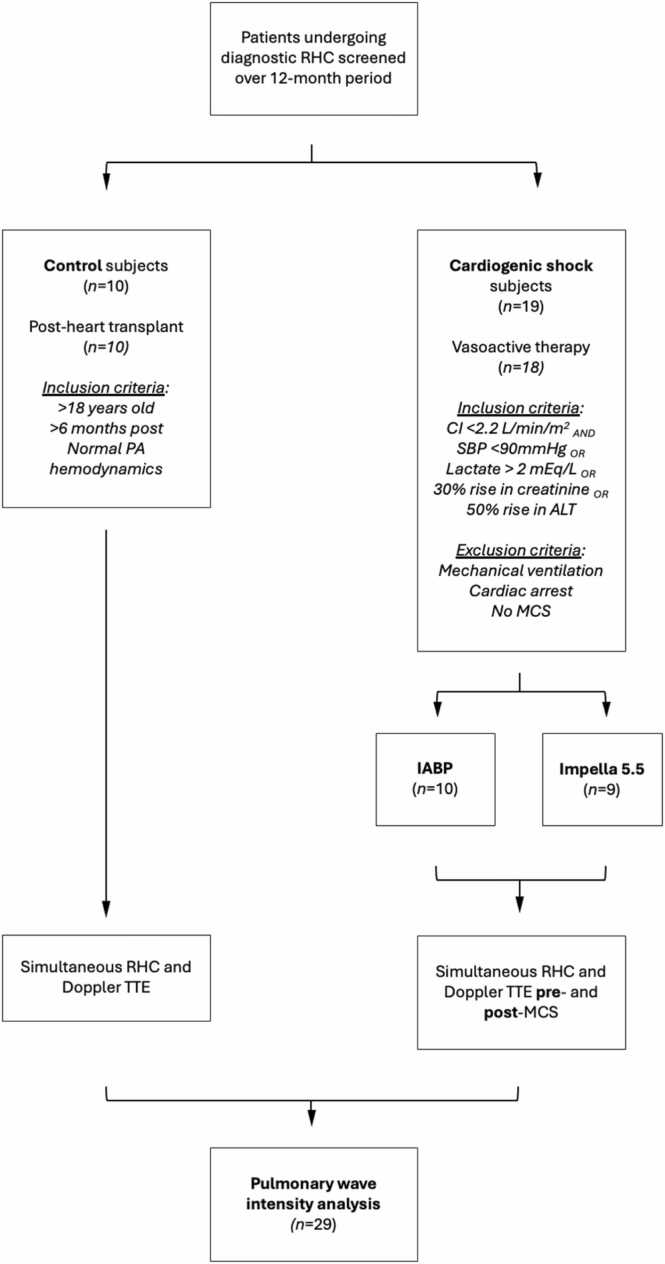
Study flow diagram. Figure caption: Study flowchart outlining patient screening and enrollment for pulmonary WIA. Over a 12-month period, patients undergoing diagnostic RHC were screened. Ten control subjects were recruited from the post-heart transplant population and were required to be >6 months post-transplant, >18 years of age, and have normal pulmonary artery hemodynamics. Nineteen patients with CS were enrolled based on low cardiac index (CI <2.2 L/min/m²) and additional markers of hypoperfusion or organ dysfunction, with exclusion of those mechanically ventilated or recently resuscitated. CS subjects underwent implantation of either an IABP or Impella 5.5 device. All patients underwent simultaneous RHC and transthoracic Doppler TTE for WIA. Control subjects had a single recording, while CS patients were assessed pre- and post-MCS device implantation. A total of 29 subjects had complete paired pressure-flow datasets available for analysis. Abbreviations: ALT**,** Alanine Aminotransferase; CI**,** Cardiac Index; CS, Cardiogenic Shock; IABP**,** Intra-Aortic Balloon Pump; Impella 5.5, Percutaneous Left Ventricular Assist Device (Abiomed Impella 5.5); MCS**,** Mechanical Circulatory Support**;** PA, Pulmonary Artery; RHC, Right Heart Catheterization; SBP, Systolic Blood Pressure; TTE**,** Transthoracic Echocardiography.

A total of 29 patients had complete RHC and transthoracic echocardiography (TTE) datasets for WIA (Figure 1). As per protocol, control subjects had simultaneous pressure-flow data collected once, while CS subjects had these data collected twice, once within 24 hours prior to MCS device implant (i.e., pre-MCS) and once within 24 hours following MCS device implant (i.e., post-MCS). Institutional Review Board was obtained for the study, and all subjects provided informed consent. Study data is available upon request from the corresponding author.

### Right heart catheterization

RHC was performed with patients fasted in the supine position using a standard 7.5 Fr Swan-Ganz catheter (Edwards Lifesciences, Irvine, CA) via the right internal jugular vein. The PA pressure waveform was obtained in the main PA ∼0.5 to 1 cm distal to the pulmonary valve and confirmed with appropriate dampening of the PA waveform. Pressure waveforms were acquired using S5 Collect software and stored with a sampling frequency of 300 Hz. Consecutive signals of PA pressure were recorded for 3-5 cardiac cycles at end-expiration.^8^, ^9^, ^10^ To remove random noise, pressure signals were smoothed by performing an ensemble average samples pressure beats as described previously.^8^, ^9^, ^10^ RHC-derived measurements included RAP, mPAP, PCWP (end-expiration), cardiac output (CO, thermodilution method), and CI.

### Transthoracic echocardiogram

TTE was performed using a EPIQ CVx Philips ultrasound machine (Philips Healthcare, Netherlands) in accordance with American Society of Echocardiography reporting guidelines. RV fractional area change (RV FAC) was measured from the apical 4-chamber view by tracing the endocardial borders at end-diastole and end-systole and expressed as a percentage change in area. End-diastolic and end-systolic RV areas (RVEDA and RVESA, respectively) were also obtained from the apical 4-chamber view. Tricuspid annular plane systolic excursion (TAPSE) was measured using M-mode echocardiography, with the cursor aligned along the lateral tricuspid annulus in the apical 4-chamber view. PASP was estimated from the peak tricuspid regurgitant jet velocity using the modified Bernoulli equation and adding the estimated RAP based on inferior vena cava size and collapsibility. Left ventricular function was evaluated by measuring LV ejection fraction (LVEF) using the biplane Simpson’s method from the apical 4- and 2-chamber views. Left ventricular end-diastolic volume (LVEDV) and LV end-systolic volume (LVESV) were also calculated using the same method. All measurements were averaged over 3 cardiac cycles for patients in sinus rhythm and over 5 cycles for those with atrial fibrillation.

### Vascular afterload

Pulmonary vascular resistance (PVR, dynes·s·cm^−5^) was estimated as *(mean PA pressure - PCWP)/*CO*.* PA compliance (PAC, mL/mmHg) was calculated as the ratio of stroke volume to pressure pulse.^11^ Derivation of impedance spectra required simultaneous measurement of PA pressure and blood flow velocity. Periodic signals of pressure and velocity were quantified in the frequency domain as a series of wave intensities (moduli) at different frequencies, known as a spectrum (i.e., the ratio of the pressure and velocity moduli at each frequency). For the pressure signal, the PA waveform was digitally scanned and the pressure values (sampled at 200 Hz) extracted using a semiautomated image analysis tool (WebPlotDigitizer v4.5). For the flow signal, the outer envelope of the pulse-wave Doppler spectrum was traced manually and the values extracted as above. The data were converted to the frequency domain using the fast Fourier transform in R statistical software (Vienna, Austria). The moduli of pressure and velocity signals from frequencies corresponding to integer multiples of the number of heart beats sampled were defined as “harmonics,” and moduli from the 0^th^ to 10^th^ harmonics were used for analysis, as increasingly higher-order harmonics largely represented signal noise. The PA impedance spectrum was then generated by dividing the pressure modulus for each harmonic by the corresponding flow modulus. Characteristic impedance (Zc) was defined as the average of the impedance moduli from the 5^th^ to 10^th^ harmonics.^9^, ^10^, ^12^, ^13^

### Wave separation and intensity analysis

Wave separation analysis was performed using the methods described by Parker et al.^1^ Briefly, pressure (P) and velocity (U) waveforms were sampled at 200 Hz using a semiautomated image analysis tool (WebPlotDigitizer v4.5). Digitized waveforms were smoothed using Savitsky-Golay filters and compared visually to the original waveforms to ensure accuracy. Multiple (typically 10-15) cardiac cycles were ensemble averaged to obtain a representative cycle.^1^ dP and dU were obtained from the second-order polynomials of the Savitsky-Golay filter and were separated into forward and backward components using the water hammer equations: *dP*_*±*_*=(dP±ρcdU)/2 and dU*_*±*_*=±(dP±ρcdU)/2ρc,* where ρc was obtained from the sum of squares method.^1^ Wave intensity, dI_±_, was calculated as the product of dP_±_ and dU_±_. Forward compression wave, decompression wave, backward compression wave (detected within 300 ms of the start of the cardiac cycle), and decompression energies were calculated as the time integral of the corresponding waves on the dI vs time plot.^1^ The time to peak backward compression wave was normalized by expressing it as a percent of the total cardiac cycle duration.^1^ The reflection coefficient was defined as the absolute value of the ratio of peak forward and backward compression wave energies, whilst net wave intensity was defined as the sum of forward and backward compression wave intensities. Wave speed was estimated using the sum of squares method: c=(1/ρ)×√[∑(ΔP)²/∑(ΔU)²]*,* where *c* is wave speed (m/s), *ρ* is blood density (typically ∼1050 kg/m³), Δ*P* is incremental pressure change, and Δ*U* is incremental flow velocity change.^14^, ^15^ High independent interobserver agreement of WIA measures from our laboratory using this methodology have been reported previously*.*^16^

### Statistics

Data analysis was performed using SPSS-27 (IBM Corporation, Armonk, New York). Normally distributed variables are presented as mean ± SD, and non-normally distributed variables as median [IQR]. Baseline characteristics were compared using the Student *t*-test for normally distributed continuous variables, the Mann-Whitney *U* test for non-normally distributed continuous variables, and the Chi-square or Fisher exact test for categorical variables. One-way ANOVA and Kruskal-Wallis tests were used to compare subgroup means across multiple groups, based on normality. Paired *t*-tests (or Wilcoxon signed-rank tests for non-parametric data) were used to assess within-subject differences in wave intensity and hemodynamic measures before and after MCS implantation in the CS cohort. Pearson and Spearman’s rho correlations were used to identify significant covariates. Generalized linear regression was performed to assess associations between WIA parameters and measures of left and right ventricular volume and function, with model fit assessed using the Wald Chi-square and Akaike Information Criterion. No formal adjustment for multiple comparisons was applied (i.e., potential for type I error). Given the modest sample size and multiple parameters, all analyses were prespecified as exploratory and hypothesis-generating. Emphasis was placed on effect sizes, consistency of paired within-subject trends, and concordance across complementary analyses (including box-and-whisker and spaghetti plots). *p* values < 0.05 were considered statistically significant.

## Results

### Baseline clinical and hemodynamic characteristics

Baseline patient characteristics are summarized in Table 1. Serum chloride and sodium were lower, and white cell count higher in subjects with CS (all *p* < 0.01). At the time of study, 8 (42%) CS patients were receiving a single vasoactive agent—either dobutamine, milrinone, or sodium nitroprusside—while 3 (16%) were on dual therapy, and 7 (37%) were receiving a combination of 3 inotropic agents. Only one patient had not yet been initiated on inotropic therapy. No patients received norepinephrine or vasopressin (Table 1).

**Table 1:** Baseline Patient Characteristics

	No CS (*n* = 10)	CS (*n* = 19)	*p-*value	Reference range
Clinical, all mean ± SD				
Age, years Sex, male Height (cm) Weight (kg)	52±16 7 (70%) 159±50 74±22	54±9 17 (89.5%) 177±52 86±21	*p* *=* 0.54 *p* *=* 0.31 *p* *=* 0.16 *p* *=* 0.48	
BSA	1.8±0.5	2.0±0.3	*p* *=* 0.30	(1.6-2.2)
Combined PH		18 (95%)		
Biomarkers				
AST, U/L ALT, U/L Blood urea nitrogen, mg/dL Creatinine, mg/dL Chloride, mmol/L Hb, g/dL Sodium, mmol/L WCC x 109/L	31±11 28±10 29±10 1.6±0.4 106±3 11.8±1.4 139±2 5.4±1.9	54±56 49±46 28±25 1.5±1.2 97±5 11.9±1.8 132±4 7.9±4.1	*p* *=* 0.68 *p* *=* 0.82 *p* *=* 0.18 *p* *=* 0.20 ***p*** ***<*** **0.01** *p* *=* 0.64 ***p*** ***<*** **0.01** ***p*** ***<*** **0.01**	(10-40) (7-56) (6-20) (0.6-1.3) (96-106) (12-16.0) (135-145) (4.0-11.0)
Inotropes				
Dobutamine Milrinone Dopamine Nor/epinephrine Vasopressin Sodium nitroprusside Nil Monotherapy Dual Triple		10 (53%) 14 (74%) 0 (0%) 0 (0%) 0 (0%) 7 (37%) 1 (5%) 8 (42%) 3 (16%) 7 (37%)		

Baseline hemodynamic and TTE characteristics are summarized in Table 2a. All measures of systemic arterial pressure were lower in subjects with CS (all *p* *<* 0.01) whilst measures of PA pressure, PVR, PAC, and Zc were higher (all *p* *<* 0.01; Table 2a). All patients with CS demonstrated significant biventricular dysfunction at baseline (LVEF 16% ± 7%; LVEDV 370 ± 183 mL; ESV of 314 ± 160 mL; all *p* *<* 0.01 vs controls). Right ventricular dysfunction was also evident (RVFAC 21% ± 11%; RVEDA 30 ± 8 cm² RVESA 24 ± 7 cm² TAPSE 1.4 ± 0.5 cm; all *p* < 0.05 vs controls; Table 2a).

**Table 2:** Baseline Hemodynamic and TTE Characteristics

a.	No CS (*n* = 10)	CS (*n* = 19)	*p* value	Reference range			
HR, bpm SBP, mmHg DBP, mmHg MAP, mmHg	84±26 130±39 80±23 96±29	97±20 97±11 66±11 76±9	*p* *=* 0.17 ***p*** ***<*** **0.01** ***p*** ***<*** **0.01** ***p*** ***<*** **0.01**	(60-100) (90-120) (60-80) (70-100)			

*LV*, all, mean ± SD							
LVEF, % LVEDV, mL LVESV, mL MV E velocity	50±15 97±34 44±17 0.7±0.3	16±7 370±183 314±160 1.8±0.7	***p*** ***<*** **0.01** ***p*** ***<*** **0.01** ***p*** ***<*** **0.01** ***p*** ***<*** **0.01**	(54-74) (76-128) (28-53) (0.6-1.3)			

*RV,* all mean ± SD							
RVFAC, % RVEDA, cm^2^ RVESA, cm^2^ TAPSE, cm RVCO, L/min RVSV, mL/beat	39±13 17±5 10±4 1.6±0.5 5.6±1.1 67±28	21±11 30±8 24±7 1.4±0.5 4.1±1.6 45±21	***p*** ***<*** **0.01** ***p*** ***<*** **0.01** ***p*** ***<*** **0.01** ***p*** = **0.04** ***p*** ***<*** **0.01** ***p*** **<** **0.01**	>35 10-24 5-16 ≥1.6 3.5-7.0 50-100			

*PA,* all mean ± SD							
CSA, cm^2^ RAP, mmHg PASP, mmHg PADP, mmHg mPAP, mmHg mPCWP, mmHg PVR, WU PAC, mL.mmHg Zc, dynes-sec/cm^5^	2.2±0.3 5±3 25±8 12±4 16±5 9±4 1.4±0.6 5.2±3.0 193±102	2.9±0.5 11±8 52±17 27±9 35±11 25±10 2.7±1.2 2.1±1.4 747±772	***p*** **<** **0.01** ***p*** ***<*** **0.01** ***p*** ***<*** **0.01** ***p*** ***<*** **0.01** ***p*** ***<*** **0.01** ***p*** ***<*** **0.01** ***p*** ***<*** **0.01** ***p*** ***<*** **0.01** ***p*** ***<*** **0.01**	3.5-7.0 2-6 15-30 4-12 9-18 6-12 0.25-2.0 2.5-5.0 N/A			

b.	IABP			Impella			
	Off (*n* = 10)	On	*p value*	Off (*n* = 9)	On	*p value*	Reference range

HR, bpm SBP, mmHg DBP, mmHg MAP, mmHg	87±23 94±8 69±13 77±10	79±19 90±12 53±11 65±9	*p* = 0.48 *p* = 0.82 ***p*** **= 0.01** ***p*** **= 0.03**	105±12 99±14 62±9 74±8	108±21 97±5 67±9 77±6	*p* = 1.00 *p* = 1.00 *p* = 0.14 *p* = 0.42	(60-100) (90-120) (60-80) (70-100)

*LV,* all, mean ± SD							
LVEF, % LVEDV, mL LVESV, mL MV E velocity	17±8 307±161 263±158 1.0±0.3	19±8 302±174 251±163 0.9±0.2	*p* = 0.79 *p* = 0.86 *p* = 0.86 *p* = 0.45	15±6 441±197 370±160 1.1±0.3	10±8 407±165 365±152 1.0±0.2	*p* = 0.19 *p* = 0.48 *p* = 0.86 *p* = 0.83	(54-74) (76-128) (28-53) (0.6-1.3)

*RV,* all mean ± SD							
RVFAC, % RVEDA, cm^2^ RVESA, cm^2^ TAPSE, cm RVCO, L/min RVSV, mL/beat	21±13 31±9 24±8 1.4±0.4 3.7±0.9 47±24	22±9 29±11 23±8 1.6±0.7 4.3±1.1 60±30	*p* = 0.86 *p* = 0.40 *p* = 0.48 *p* = 0.69 *p* = 0.20 *p* = 0.34	21±8 30±8 23±7 1.8±0.6 4.6±1.2 45±17	23±9 31±6 24±7 1.6±0.5 4.7±2.0 45±19	*p* = 0.60 *p* = 0.33 *p* = 0.48 *p* = 0.66 *p* = 0.83 *p* = 0.93	>35 10-24 5-16 ≥1.6 3.5-7.0 50-100

*PA,* all mean ± SD							
CSA, cm^2^ RAP, mmHg PASP, mmHg PADP, mmHg mPAP, mmHg mPCWP, mmHg PVR, WU PAC, mL.mmHg Zc, dynes-sec/cm^5^	2.8±0.6 12±8 50±16 24±8 32±11 23±9 2.8±1.3 2.3±2.0 725±592	2.8±0.6 13±6 45±12 23±7 31±8 22±7 2.2±1.1 3.0±2.1 783±931	*p* = 0.97 *p* = 0.79 *p* = 0.31 *p* = 0.69 *p* = 0.53 *p* = 0.65 *p* = 0.37 *p* = 0.48 *p* = 0.88	2.9±0.4 11±8 55±18 30±10 38±12 27±11 2.4±1.2 2.1±1.5 770±956	2.8±0.5 9±4 55±18 28±8 37±11 22±9 3.2±1.5 1.9±0.5 387±258	*p* = 0.67 *p* = 0.79 *p* = 0.92 *p* = 0.81 *p* = 0.85 *p* = 0.30 *p* = 0.35 *p* = 0.65 *p* = 0.09	3.5-7.0 2-6 15-30 4-12 9-18 6-12 0.25-2.0 2.5-5.0 N/A

Hemodynamic and transthoracic echocardiographic (TTE) responses to MCS, stratified by device type, are summarized in Table 2b. Conventional hemodynamic and TTE indices changed minimally in the acute post-MCS state. Aside from a reduction in diastolic blood pressure (DBP, *p* = 0.01) and mean arterial pressure (MAP, *p* = 0.03), no significant between-group differences in PA hemodynamic or TTE parameters between the IABP-off and IABP-on states (all *p* > 0.05) were observed. Similarly, there were no significant hemodynamic or TTE differences between the Impella-on and Impella-off groups (all *p* > 0.05; Table 3).

**Table 3:** WIA Parameters in Controls Versus CS Patients Pre- and Post-MCS

a.	All control (*n* = 10)	CS (Pre-MCS) (*n* = 19)	*p* value	Post MCS (*n* = 19)	*p* value pre- vs post- MCS	*P* value control vs post- MCS
Forward						
FCW, mmHg.cm/s FDW, mmHg.cm/s	0.12±0.03 0.05±0.02	0.14±0.06 0.08±0.10	*p* = 0.16 *p* = 0.08	0.21±0.14 0.12±0.07	*p* = 0.33 *p* *=* 0.34	***p*** **=** **0.02** ***p*** ***<*** **0.01**

Reflected						
BCW, mmHg.cm/s TTP, s BDW, mmHg.cm/s NWI RI Wave speed	−0.04±0.02 0.18±0.07 −0.01±0.01 −0.02±0.02 0.36±0.39 1.8±1.0	−0.08±0.09 0.13±0.07 −0.03±0.02 −0.05±0.07 0.54±0.48 7.1±7.4	*p* = 0.06 *p* = 0.09 ***p*** < **0.01** ***p*** = **0.03** *p* = 0.29 ***p*** ***<*** **0.01**	−0.07±0.07 0.17±0.05 −0.03±0.03 −0.04±0.05 0.49±0.48 5.6±6.6	*p* = 0.75 ***p*** ***=*** **0.048** *p* = 0.72 *p* *=* 0.27 *p* = 0.72 *p* *=* *0.38*	*p* = 0.06 *p* = 0.66 ***p*** **< 0.01** *p* = 0.54 *p* = 0.45 ***p*** **= 0.03**

b.	IABP Off (*n* = 10)	On	*p* value	Impella Off (*n* = 9)	On	*p value*

Forward						
FCW, mmHg.cm/s FDW, mmHg.cm/s	0.14±0.07 0.06±0.13	0.20±0.11 0.10±0.04	*p* = 0.08 ***p*** **= 0.04**	0.19±0.17 0.13±0.13	0.22±0.17 0.14±0.13	*p* = 0.86 *p* = 0.87

Reflected						
BCW, mmHg.cm/s TTP, s BDW, mmHg.cm/s RI NWI Wave speed	−0.11±0.10 0.16±0.07 −0.03±0.03 0.76±0.50 −0.09±0.09 6.9±5.6	−0.08±0.08 0.19±0.07 −0.03±0.02 0.42±0.33 −0.05±0.07 7.5±8.7	*p* = 0.21 *p* = 0.38 *p* = 0.93 ***p*** **= 0.046** *p* = 0.14 *p* = 0.83	−0.04±0.03 0.10±0.05 −0.03±0.02 0.26±0.33 −0.02±0.03 8.1±9.4	−0.07±0.06 0.15±0.04 −0.04±0.05 0.58±0.63 −0.03±0.03 4.0±2.4	*p* = 0.22 ***p*** = **0.03** *p* = 0.69 *p* = 0.30 *p* = 0.77 *p* = 0.21

Abbreviations: BCW, backward compression wave; BDW, backward decompression wave; CO, cardiac output; CSA, cross-sectional area; EDV, end diastolic volume; ESV, end systolic volume; EF, ejection fraction; FCW, forward compression wave; FDW, forward decompression wave; mPAP, mean pulmonary artery pressure; mPCWP, pulmonary capillary wedge pressure; PAC, pulmonary arterial compliance; PVR, pulmonary vascular resistance; RI, reflection index; RAP, right atrial pressure; RAV, right atrial volume; RV, right ventricular; SV, stroke volume; SWi, stroke work indexed; TTP, time to peak; Zc, characteristic impedance.

### Characterization of wave propagation

**Figure 2:**
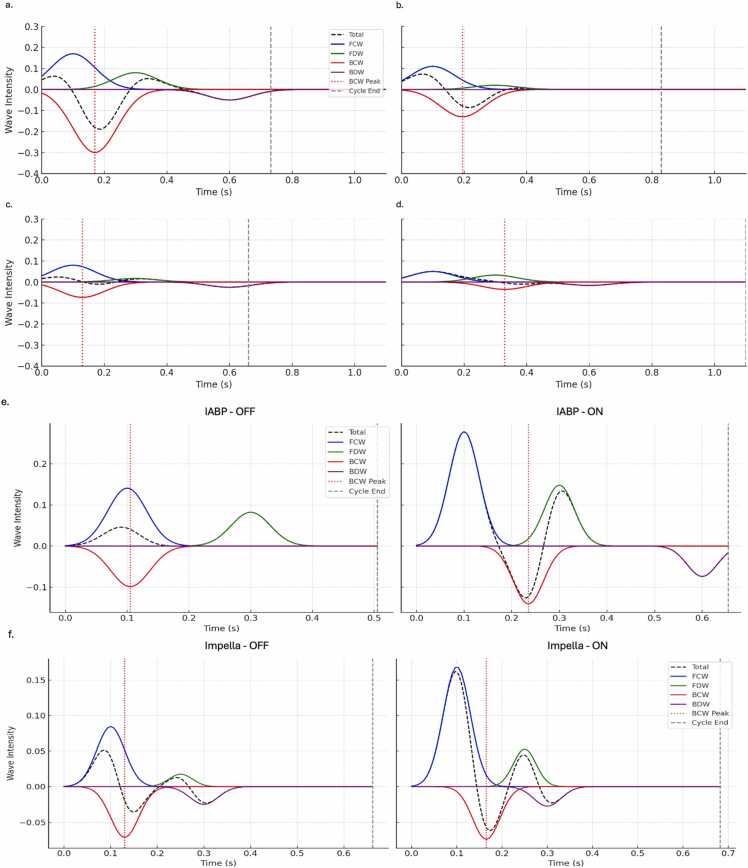
Representative pulmonary wave intensity analyses. Figure caption: Each panel displays wave intensity profiles derived from pressure-flow data over a single cardiac cycle, showing forward compression waves (FCW, blue), forward decompression waves (FDW, green), backward compression waves (BCW, red), and backward decompression waves (BDW, purple). The timing of peak BCW (red dotted line) and end of the cardiac cycle (black dotted line) are marked. All plots are normalized to the same *x*- (time) and *y*-(wave intensity) axes for comparison. 1a: pulmonary WIA during dobutamine infusion at 7.5 µg/kg/min in a patient with CS. This high-dose inotropic support is associated with increased forward wave intensity, a moderate increase in backward wave intensity, but no increase in reflected wave speed. 1b: pulmonary WIA during milrinone infusion at 0.375 µg/kg/min in a patient with CS. There is a minimal increase in forward-traveling wave intensity with salutatory effects on the backward-traveling wave intensity. There is a relative reduction in reflected wave speed, likely due to peripheral vasodilation. 1c: pulmonary WIA during sodium nitroprusside infusion at 1.25 µg/kg/min. There is minimal effect on forward or backward-traveling wave intensities. Reflected wave speed is increased consistent, likely due to the systemic vasodilatory action of sodium nitroprusside without direct inotropic effect. 1d: pulmonary WIA from a patient in CS prior to any treatment initiation. There are markedly reduced forward and backward wave intensities, with late return of the reflected wave, suggesting blunted wave generation and delayed reflection timing, likely due to CS with relative vasoplegia. 1e: pulmonary WIA from a patient in CS with IABP support-off and IABP support-on. IABP-on support is associated with increased forward-traveling wave intensities, as well as a delay to peak backward compression wave. 1f: pulmonary WIA from a patient in CS with Impella support off and Impella support on (performance level 8, flow 4.8 L/min). Impella-on support is also associated with increased forward-traveling wave intensities, with no significant effect on backward-traveling wave magnitudes or timing. Abbreviations: BCW, backward compression wave; BDW**,** backward decompression wave; CS**,** cardiogenic shock; FCW**,** forward compression wave; FDW**,** forward decompression wave; IABP**,** intra-aortic balloon pump; Impella, percutaneous left ventricular assist device (Abiomed Impella 5.5); MCS, mechanical circulatory support; WIA**,** wave intensity analysis.

Representative pulmonary wave intensity analyses are presented in Figure 2. All 29 patients (100%) exhibited a dominant forward compression wave, forward decompression wave, backward compression wave, and backward decompression wave at baseline. The backward decompression wave disappeared in 1 CS subject with Impella 5.5 insertion.

**Figure 3:**
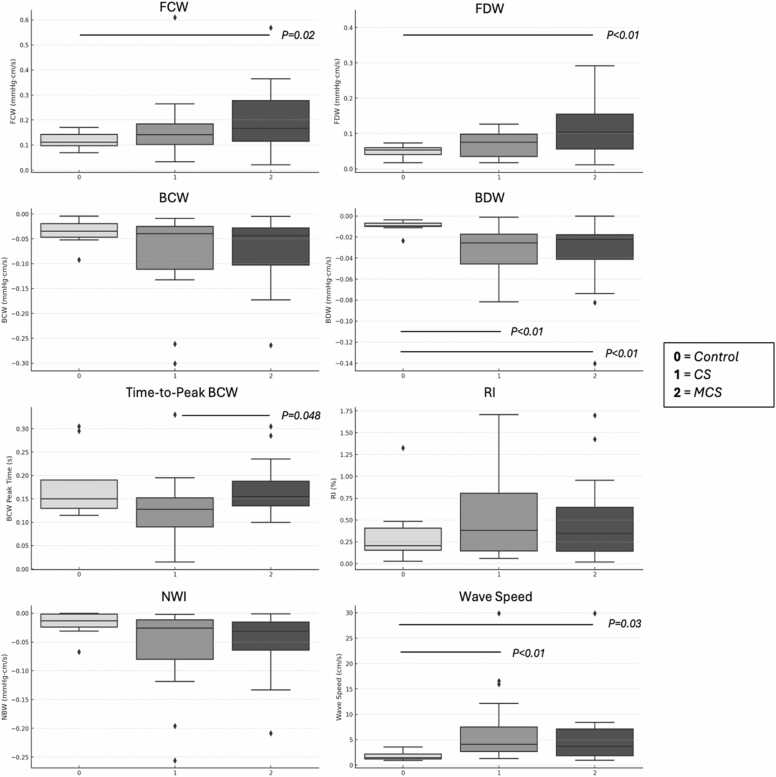
Box-and-Whisker plots. Figure caption: Box-and-whisker plots demonstrate forward compression wave (FCW), forward decompression wave (FDW), backward compression wave (BCW), backward decompression wave (BDW), time-to-peak BCW, reflection index (RI), net wave intensity (NWI), and wave speed in 3 groups: healthy controls (0), patients with cardiogenic shock (CS; 1), and those supported with mechanical circulatory support (MCS; 2) using either intra-aortic balloon pump (IABP) or Impella 5.5. Significant pairwise differences were observed between controls and CS for BDW (*p* < 0.01) and wave speed (*p* < 0.01), and between CS and MCS for time-to-peak BCW (*p* = 0.048). Significant pairwise differences between controls and MCS subjects in FCW (*p* = 0.02), FDW (*p* < 0.01), BDW (*p* < 0.01), and wave speed (*p* = 0.03) were observed. Abbreviations: BCW, backward compression wave; BDW**,** backward decompression wave; CS**,** cardiogenic shock; FCW**,** forward compression wave; FDW**,** forward decompression wave; IABP**,** Intra-Aortic Balloon Pump; Impella**,** Percutaneous Left Ventricular Assist Device (Abiomed Impella 5.5); MCS, mechanical circulatory support; NWI, net wave intensity; WIA**,** wave intensity analysis.

**Figure 4:**
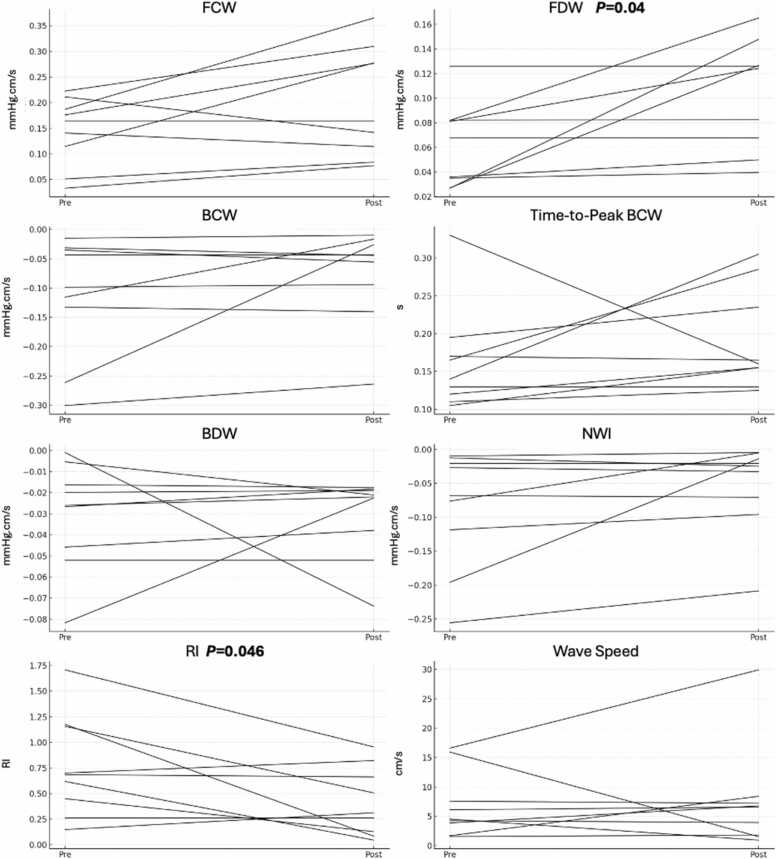

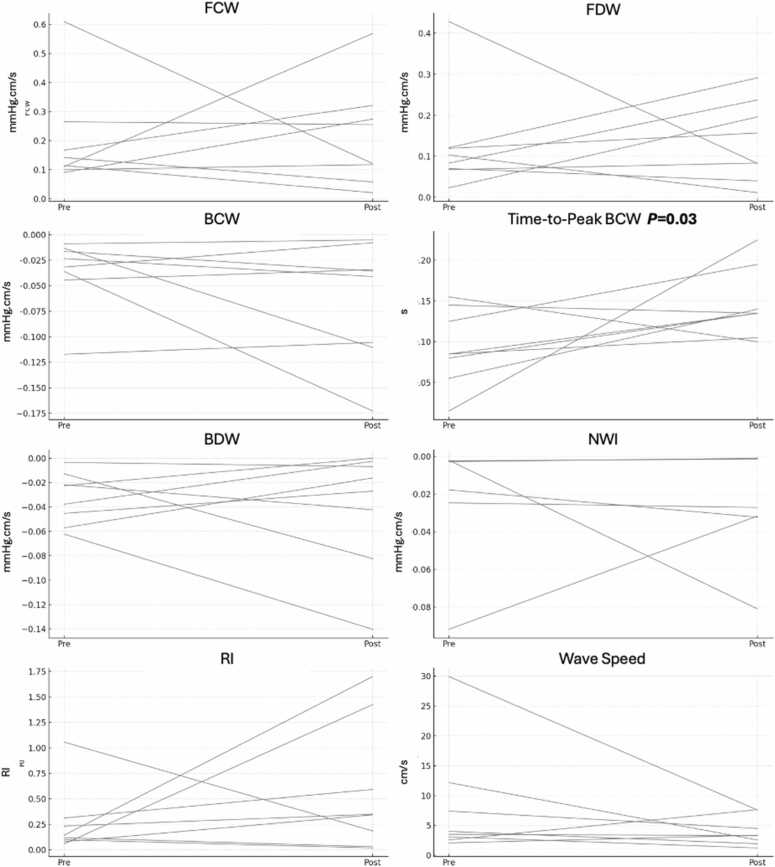
Spaghetti plots pre- and post- MCS intervention. 4 (top): Pre- and post-IABP support changes in wave intensity parameters among patients with CS. Forward wave magnitudes (FCW, FDW) increased in most patients, while backward wave magnitudes (BCW, BDW) remained largely unchanged, resulting in a consistent reduction in reflection index (RI). There was no significant change in time-to-peak BCW or wave speed, suggesting preserved timing and stiffness characteristics. A general upward shift in net wave intensity (NWI) was observed, indicating improved overall wave energy balance and potentially more favorable ventriculo-arterial coupling with IABP support. 4 (bottom): Pre- and post-Impella 5.5support changes in wave intensity parameters among patients with CS. Following Impella 5.5 insertion, FCW and FDW magnitudes increased in several patients, though a subset showed either no change or a decrease. BCW timing was consistently delayed post-support, indicating later arrival of reflected waves. Backward wave magnitudes (BDW and BCW) generally became less negative, reflecting a reduction in wave reflection severity, although exceptions were noted. These changes were associated with a notable increase in RI in most individuals. Net wave intensity shifted upward in some cases, but responses were heterogeneous. Wave speed showed no consistent pattern, with both increases and decreases observed across the cohort. Abbreviations: BCW, backward compression wave; BDW**,** backward decompression wave; CS**,** cardiogenic shock; FCW**,** forward compression wave; FDW**,** forward decompression wave; IABP**,** Intra-Aortic Balloon Pump; Impella**,** Percutaneous Left Ventricular Assist Device (Abiomed Impella 5.5); MCS, mechanical circulatory support; WIA**,** wave intensity analysis.

WIA parameters are summarized in Table 3. Box and whisker plots are presented in Figure 3. Sphagetti plots pre- and post-MCS are presented in Figure 4. All comparisons that reached nominal statical significance were interpretated as exploratory given the modest sample size. Forward compression (0.12 ± 0.07 vs controls 0.05 ± 0.02 mmHg.cm/s) and decompression (0.12 ± 0.07 vs controls 0.05 ± 0.02 mmHg.cm/s) wave magnitudes were highest in patients in the post-MCS state (both *p* *<* 0.05). There were no significant between group differences in backward compression wave magnitude (all pairwise comparisons *p* > 0.05), however backward decompression wave magnitude was more negative in patients with CS (−0.03 ± 0.02 mmHg·cm/s) in the post-MCS state (−0.03 ± 0.03 vs controls −0.01 ± 0.01 mmHg·cm/s; both pairwise comparisons *p* < 0.01), as was net wave intensity (−0.05 ± 0.07 vs controls −0.02 ± 0.02 mmHg·cm/s; *p* = 0.03) in CS subjects. Wave speed was increased in subjects with CS (7.1 ± 7.4 vs controls 1.8 ± 1.0 m/s; *p* < 0.01). No between group differences in backward compression wave magnitude and/or timing were observed (all pairwise comparisons *p* > 0.05).

### Correlation with vascular afterload

Correlation analysis was performed to evaluate associations between wave intensity metrics and established pulmonary vascular load parameters, including PVR, PAC, and Zc. All associations were modest in magnitude and interpreted as hypothesis-generating. Forward compression decompression wave magnitude demonstrated moderate inverse correlations with PAC (*r* = −0.39 for both) and positive correlations with PVR (*r* = 0.45 and *r* = 0.26, respectively), indicating reduced PAC and elevated PVR were associated with attenuated forward-traveling waveforms. Conversely, backward decompression wave magnitude was positively correlated with PAC (*r* = 0.44) and inversely correlated with PVR (*r* = −0.43). Net wave intensity demonstrated a strong inverse correlation with PVR (*r* = −0.43) and a positive correlation with PAC (*r* = 0.44). The backward compression wave and reflection index showed weaker, more variable correlations with all vascular load parameters (all *p* *>* 0.05). Backward compression wave peak timing was not meaningfully associated with any hemodynamic variable (all *p* *>* 0.05).

### Regression analyses

Univariable linear regression identified wave intensity-derived parameters as optimal correlates for key LV/RV parameters, though limited sample size and multiple regression models used mean all associations should be interpreted as exploratory (Table 4). Although PVR was the strongest correlate of LVEF (*R*² = 0.19, *p* < 0.01), backward decompression wave intensity emerged as the best predictor of both LVEDV (*R*² = 0.13, *p* = 0.02) and LVESV (*R*² = 0.13, *p* = 0.02), with higher wave magnitudes associated with smaller LV volumes. For left atrial volume, the strongest association was observed with backward compression wave intensity (*R*² = 0.23, *p* *<* 0.01). Right ventricular function, indexed by RVFAC, was most closely related to PVR (*R*² = 0.28, *p* *<* 0.01); however, regression analysis also showed that backward compression wave intensity was inversely associated with both RVEDA (*R*² = 0.31, *p* *<* 0.01) and RVESA (*R*² = 0.24, *p* *<* 0.01), while no significant association was observed with TAPSE (*p* = 0.46). Wave intensity measures also correlated with the dose requirements for vasoactive therapy and Impella 5.5 performance level. Net wave intensity was the strongest predictor of dobutamine dose (*R*² = 0.30, *p* = 0.02), with lower net wave intensity associated with higher dosage. Milrinone dose showed the strongest inverse association with backward compression wave peak time (*R*² = 0.37, *p* *<* 0.01). Impella 5.5 performance level was most strongly predicted by net wave intensity (*R*² = 0.91, *p* *<* 0.01).

**Table 4:** Linear Regression Analysis

Outcome	Best predictor	*R*²	Beta	*p*	*R*² (PVR)	Beta (PVR)	*p* (PVR)
Left ventricle							
LVEF, %	PVR	0.186	−0.06	0.0062	0.186	−0.06	0.01
LVEDV, mL	BDW	0.131	−2922.94	0.0237	0.09	41.29	0.06
LVESV, mL	BDW	0.133	−2873.65	0.0226	0.109	44.46	0.04
LAV, mL	BCW	0.234	−143.66	0.0024	0.164	6.77	0.01

Right ventricle							
RVFAC, %	PVR	0.282	−0.05	0.0004	0.282	−0.05	<0.01
RVEDA, cm^2^	BCW	0.311	−66.29	0.0002	0.029	1.14	0.29
RVESA, cm^2^	BCW	0.237	−52.82	0.0014	0.131	2.2	0.02
TAPSE, cm	BCW	0.083	−20.59	0.0796	0.015	0.5	0.46

Clinical markers							
Lactate	PVR	0.15	0.1	0.1122	0.15	0.1	0.11
ALT	PVR	0.013	−1492.46	0.4876	0.013	−1492.46	0.49
Dobutamine, mg	NWI	0.298	−13.4	0.0233	0.002	−0.06	0.86
Milrinone, mg	TTP	0.374	−1.16	0.0019	0.047	−0.02	0.32
Impella, P level	NWI	0.908	38.27	0.0032	0.086	0.16	0.57

Abbreviations: BCW, backward compression wave; FCW, forward compression wave; FDW, forward decompression wave; mPAP, mean pulmonary artery pressure; PVR, pulmonary vascular resistance; RI, reflection index; RVCO, right ventricular cardiac output; RVEDV, right ventricular end-diastolic volume; RVEF, right ventricular ejection fraction; RVESV, right ventricular end-systolic volume; RVSWi, right ventricular stroke work index.

## Discussion

This study represents the first known investigation of pulmonary WIA in patients with CS undergoing MCS. Our findings can be summarized as follows: (1) In the setting of CS, net wave intensity becomes more negative due to a relative reduction in forward compression wave magnitude, while backward compression wave magnitude remains unchanged or increases slightly; (2) Wave speed is significantly elevated in patients with CS; (3) MCS is associated with enhancement of forward-traveling wave magnitudes and delays the return of the backward compression wave; (4) IABP support increases forward decompression wave magnitude and reduces reflection index, whereas Impella 5.5 support primarily delayed backward wave return, and (5) WIA-derived metrics appeared to detect physiologic changes in RV-PA coupling that were not consistently reflected by conventional hemodynamic indices such as mPAP or PVR.

### Historical perspectives in context

WIA, as applied to the systemic circulation, was first described by Parker et al in 1990.^1^ More recently, Yim et al evaluated pulmonary WIA in patients with PH due to left-sided heart disease (where CS was excluded).^5^ In *Yim’s* study, mean LVEF was 21% ± 14% and RVFAC was 26% ± 12%. All patients (100%) exhibited a forward compression wave associated with RV contraction and a forward decompression during diastole. The presence of backward compression and decompression waves was detected in 70% and 55% of patients, respectively. In contrast, our study extends these findings to a critically ill CS population for the first time. Subjects with CS universally (100%) exhibited both a backward compression and decompression wave. This supports the notion that the backward compression wave may serve as a marker of adverse pulmonary vascular hemodynamics and is reinforced by prior studies which show the backward compression wave to disappear after initiation of dobutamine in PH due to left-sided disease (with no new development of a backward compression wave in those who did not have 1 prior).^5^ The presence of a backward compression wave has also been associated with more severe heart failure (as indicated by significantly lower CO and CI, elevated PA pressures, reduced TAPSE, TAPSE/PASP ratio, and decreased arterial compliance).^5^

### Pulmonary vascular afterload

In prior studies of patients with PH from left-sided disease, WIA has been shown to correlate with mPAP, but not PVR or PAC.^15^ Reflection index however, had been previously related to PVR.^15^ In the present study of subjects with CS, reduced PAC and elevated PVR were associated with attenuated forward-traveling waveforms. Conversely, both net wave intensity and backward decompression wave magnitude were positively associated with PAC and inversely correlated with PVR. The latter is consistent with the purported role of backward decompression waves in beneficially decreasing pressure whilst increasing velocity. Further, as PCWP increased, so too did the magnitude of the backward compression wave. Although these relationships were modest in strength and should be interpreted cautiously given the limited sample size, they are nevertheless in keeping with prior analyses of pooled data with rest and dobutamine challenge that have observed a correlation between backward compression wave magnitude and PCWP.^5^

### Inotropic and device effects

There has been limited study of the effect of inotropy on wave propagation in the PA of humans, and none with subjects on milrinone. In those with PH due to left-sided HF, dobutamine is known to have salutary effects on the pulmonary vascular afterload with the benefits of RV and LV chronotropy. Dobutamine challenge with 20 mcg/kg/min has previously been observed to increase forward compression wave intensity with no effect on backward compression wave intensity, reflection index, or wave speed.^5^ In the present study, patients with lower net wave intensity required higher doses of dobutamine, suggesting net wave intensity may reflect greater baseline contractile dysfunction in patients requiring higher pharmacologic support. Higher doses of milrinone were associated with later return of the backward compression, in keeping with what is known about the vasodilatory effects of this medication. Importantly, these associations reflect treated CS physiology and cannot be interpreted as causal effects of specific pharmacologic agents.

No prior studies have applied WIA in patients receiving MCS. In this cohort, conventional hemodynamic and echocardiographic measures showed minimal change after device activation, except for a slight fall in diastolic and mean arterial pressure in IABP users. In contrast, WIA revealed clear physiologic shifts: MCS increased forward-traveling and backward decompression wave magnitudes, delayed backward compression wave return, and modestly reduced wave speed. These findings were observed at the group level when comparing pre- and post-device measurements within the same CS cohort and should again be interpreted as exploratory. IABP enhanced forward decompression wave magnitude and lowered reflection index, while the Impella 5.5 primarily delayed backward wave return. These device-specific patterns are biologically plausible given their differing mechanisms and anatomical locations, but the small sample size limits definitive comparisons between devices. Further, while these patterns are physiologically consistent with improved VA coupling, the concurrent use of vasoactive therapy and clinical heterogeneity of the study cohort limits attribution of these changes to device effects alone. Nevertheless, these changes likely reflect the integrated effects of disease severity, pharmacologic support, and mechanical unloading rather than isolated device effects.

### Clinical implications

The findings from this study suggest several potential clinical applications for pulmonary WIA in the management of CS with MCS. Given the observed associations between WIA metrics, cardiac volumes, and function, pulmonary WIA may serve as a non-invasive, real-time tool to monitor cardiac performance and detect early structural changes in patients receiving vasoactive therapy or MCS. This could support earlier risk stratification and inform timely, targeted interventions to preserve ventricular function. While WIA appeared more sensitive than conventional indices for detecting acute physiologic changes after device initiation, the present study was not designed to establish prognostic significance or to define device-specific therapeutic targets. Notwithstanding, these findings highlight the potential of WIA to detect dynamic changes in RV-PA coupling following MCS that may not be captured by standard hemodynamic measures. Future studies with larger cohorts and prospective stratification by pharmacologic and device class are required to validate these preliminary observations, as well as define normative WIA values, establish optimal diagnostic thresholds, and validate clinical cut-offs for prognostic and therapeutic use.

### Limitations

To our knowledge, this is the first cohort of patients with CS undergoing MCS to undergo pulmonary WIA. However, several limitations warrant consideration. The control group comprised clinically stable post-heart transplant recipients rather than healthy volunteers; although practical for invasive comparison, transplant-related factors and immunosuppression may influence pulmonary vascular properties. The CS cohort was small and clinically heterogeneous, with most patients receiving vasoactive therapy at the time of assessment. Consequently, the observed wave patterns reflect treated CS physiology. The study was not powered to perform stratified analyses by pharmacologic class, particularly given frequent combination therapy, and associations between WIA parameters and drug dose should be interpreted as descriptive rather than causal. MCS devices were also heterogeneous; IABP and Impella differ in mechanism and anatomical location, and device-specific findings should be considered hypothesis-generating. Given the exploratory design, multiple parameters were examined without formal adjustment for multiple comparisons, increasing the risk of type I error. In addition, individual outliers may have influenced group estimates in this modest sample. Pulmonary WIA was derived from fluid-filled catheter recordings, which may introduce signal distortion, and post-MCS assessments were limited to the acute phase. Validation in larger, prospectively designed cohorts with standardized pharmacologic and device protocols is required.

## Conclusions

In CS, pulmonary WIA demonstrated a reduction in net wave intensity driven by diminished forward compression wave magnitude, along with elevated wave speed consistent with increased pulmonary vascular stiffness. Initiation of MCS was associated with enhanced forward wave magnitude and delayed return of reflected waves, suggesting alterations in RV-PA coupling. WIA parameters were associated with structural and functional cardiac measures and appeared to reflect physiologic changes not consistently captured by conventional hemodynamic or echocardiographic indices. These findings were exploratory and hypothesis-generating and warrant validation in larger prospective studies to determine their clinical utility.

## Financial support

Dr Hungerford National Heart Foundation of Australia Post-Doctoral Fellowship and Vanguard Award. Dr Gulati Tufts CTSI Pilot Studies Award.

## Conflicts of Interest statement

The authors declare the following financial interests/personal relationships, which may be considered as potential competing interests: Sara Hungerford reports financial support was provided by the National Heart Foundation of Australia. If there are other authors, they declare that they have no known competing financial interests or personal relationships that could have appeared to influence the work reported in this paper.
